# Testing the validity of a stage assessment on health enhancing physical activity in a chinese university student sample

**DOI:** 10.1186/s12889-016-2931-2

**Published:** 2016-03-15

**Authors:** Yanping Duan, Sonia Lippke, Ru Zhang, Walter Brehm, Pak-Kwong Chung

**Affiliations:** Department of Physical Education, Hong Kong Baptist University, Hong Kong, China; Bremen International Graduate School of Social Sciences (BIGSSS), Jacobs University Bremen, Bremen, Germany; Institute of Exercise and Public Health, University of Leipzig, Leipzig, Germany; Institute of Sport Science, University of Bayreuth, Bayreuth, Germany; Department of Psychology & Methods, Jacobs University Bremen, Campus Ring 1, 28759 Bremen, Germany

**Keywords:** Stages, Physical activity, Misclassification, Specificity, Sensitivity

## Abstract

**Background:**

The study examined the measurement quality of a stage algorithm measuring the Four steps from Inactivity to activity Theory (FIT Model).

**Methods:**

In a cross-sectional study, stages were assessed in 1012 Chinese university students in terms of physical activity, social-cognitive variables and health outcomes. Main outcome measures were stages of change, self-reported physical activity, perceived barriers, intrinsic motivation, plans, fitness and health satisfaction. Misclassification, sensitivity, specificity, receiver operating characteristic (ROC) curves, nonlinear trends, and planned comparison were computed.

**Results:**

Compared to previous studies, sensitivity was at the average level (64 %-71 %), and specificity was comparably higher (76%-89%). When using higher PA intensity criteria (moderate and strenuous intensities), sensitivity was higher, whereas specificity was lower in comparison to the lower PA intensity criteria (also including mild activity). After running contrast and trend analyses, nonlinear trends for all indicative variables across the stages and a match of 77 % of predictions of stage differences were confirmed.

**Conclusion:**

The measurement quality of the stage algorithm was supported in a young adult sample.

## Background

Health enhancing physical activity (HEPA) is a primary avenue for improving physiological and mental health. The recommendations for HEPA depend on population age [1], and it is proposed that 240 min of moderate intensity physical activity (PA) per week may be an adequate recommendation for people undergoing the transition from adolescence to adulthood [2]. University students are a special group in this transition period as they show an increasing nonadherence with this recommendation [3]. Approximately half of university students do not engage in the recommended 240 min of physical activity per week [4]. The adoption and maintenance of such a recommendation is a major public health concern. From a population perspective, a larger proportion of these individuals should adhere to the recommendation, hence, a better understanding of this phenomenon is required. The current study, therefore, focused on a theoretical model which is described below, and was sought to test it in a population-based sample.

According to stage models, health behavior change is a dynamic process that consists of a sequence of discrete and qualitatively distinct stages [5]. These stages represent states through which individuals pass towards the adoption of the target behavior (e.g., 240 min of moderate intensity physical activity per week). While a developmental approach would follow-up on individuals passing through those stages, a public health approach would compare people within one stage with those in adjacent stages. Such a public health approach has the advantage of examining larger groups of individuals i.e. following a population approach. It has, thus, been selected to assess those stages and to compare the different groups. While this was done in previous research, little attention was given to the university student population with regards to the recommendation to perform HEPA that is relevant to public health and also taking into account unstable habits such as fluctuation. Thus, the current study aims at closing this gap.

Based on the previous models (Transtheoretical Model, TTM; Health Action Process Approach, HAPA [6, 7]), the recently developed *Four steps from Inactivity to activity Theory* (FIT Model) was specifically designed to describe and explain HEPA behavior [2, 8]. It integrates all five stage classifications of the TTM, but adds a “Fluctuation” stage based on evidence supporting the existence of such a stage of instability [9, 10]. In addition, the time frame of 12 months for engaging PA in the FIT model is twice as long as in the TTM. The rationale is based on previous research regarding adherence to the HEPA recommendation [11]. With that, the innovative and important purpose of this study is to test the HEPA in a student population while taking into account their lifestyle.

The FIT model comprises a series of six stages from lower to higher levels: (a) *Not-Considering*, not thinking about becoming physically active in the future; (b) *Considering*, thinking about becoming physically active soon; (c) *Preparing*, making decisions and building up plans to start physical activity soon; (d) *Fluctuating,* becoming physically active often, but not regularly every week; (e) *Exploring*, becoming physically active regularly for fewer than 12 months; and (f) *Maintaining,* becoming regularly physically active for 12 months or longer. Among these six stages, the former three are regarded as inactive stages while the latter three are active stages. Other constructs in the FIT model are (1) self-reported PA behavior including frequency, intensity and type; (2) ten social-cognitive correlates, which are essential for the transition between stages; and (3) five health outcomes associated with the stages of change (for the detailed description, see [8]).

Concurrent validity of the FIT model has generally been supported by findings that individuals in earlier stages of change participate in less PA than those more likely to adhere to the recommendation [12, 13]. Social-cognitive variables that influence the probability of moving to the next stage can be compared across the stages, which is regarded as a useful approach to test the notion of distinct stages [14, 15]. Also, measures of health outcome variables are associated with stages [8]. In other words, the more a person is progressing in the stages of physical activity adoption, the greater the health benefits a person gains [16, 17].

Differences in health outcomes between non-active stages and active stages should be evident if stage classifications are valid. Such validations of FIT model stages have been preliminarily conducted across young adults and adults both in Germany and in China [2, 8]. These studies revealed that barriers, intrinsic motivation and plans were the critical social-cognitive variables related to the FIT stages. Furthermore, the link between the FIT stages and health outcomes, including fitness and health satisfaction, was well established. However, there has been no in-depth study to comprehensively evaluate the stage validity of the FIT model until now. Therefore, the main focus of this study was to evaluate the measurement qualities of this stage-algorithm on the basis of the FIT model among university students, who are in the transition period from adolescence to adulthood. In addition, PA behavior, barriers, intrinsic motivation, plans, fitness and health satisfaction were employed as test variables.

### Examining the measurement quality of the stage algorithm

One method of evaluating measurement quality is to examine the percentages of misclassification in respect to behavior. This approach combines the three "inactive" stages (Not-Considering, Considering, Preparing) and the three "active" stages (Fluctuating, Exploring and Maintaining), and they are compared between each other on goal behavior. The comparison focuses on two aspects of classification accuracy [18, 19] (see Table 1): *Sensitivity* (agreement between classification as being active by the stage algorithm, and performing the goal activity according to a different criterion measure) and *Specificity* (agreement between the classification as being inactive, and the nonperformance of the goal activity).

**Table 1:** Computing sensitivity and specificity of stages of change for Physical Activity (PA)

Stage algorithm	Actual physical activity criterion	
	Individuals meeting physical activity criterion	Individuals not meeting physical activity criterion
Individuals in active stages (F, E, Ma)	a	b
Individuals in inactive stages (NC, C, P)	c	d

Note: NC = Not-considering; C = Considering; P = Preparing; F = Fluctuating; E = Exploring; Ma = Maintaining. *Sensitivity* = a/(a + c) = Proportion of individuals who are accurately classified as performing the activity over the total number who meet the criteria for the activity. *Specificity* = d/(b + d) = Proportion of individuals who are accurately classified as not performing the activity over the total number who don't meet the criteria for the activity

The few studies studying specificity and sensitivity of stage algorithms on PA have shown dependency on PA intensity. In a review of over 30 studies using TTM stage definitions, Nigg [20] reported that overall, the sensitivity for individuals engaging in strenuous, moderate and mild PA was 86, 71 and 54 % respectively, while specificity was 71, 63 and 47 % respectively. By testing a refined TTM stage algorithm, Lippke et al. [21] found that for strenuous and moderate activity measures, sensitivity and specificity were both 80 %. If mild activities were included in the activity assessment, measurement qualities decreased to a sensitivity of 65 %, with a specificity of 81 %. Another study addressing a three-stage HAPA algorithm revealed that for different rehabilitation patients, sensitivity and specificity on strenuous and moderate activity measure were 73 to 77 % and 87 to 88 % respectively; while sensitivity and specificity on all three intensities measures were 44 to 83 % and 70 to 79 % respectively [19]. However, no specificity and sensitivity estimates have thus far been reported for a six-stage FIT algorithm in university student samples. Therefore, the current study aimed to close this gap, and better understand and support this specific target group.

### Testing stage assumptions: nonlinearity

Previous authors [e.g., 10, 14] suggested investigating whether a stage model is actually better than a continuous model by testing for discontinuity patterns in terms of nonlinear trends. A linear trend would produce a series of ordered significant differences between stages (e.g., Not-Considering < Considering < Preparing < Fluctuating < Exploring < Maintaining). Such a linear trend would rather disprove a stage model [14, 21, 22]. In contrast, a nonlinear trend is indicated not only by significant increases in means of test variables but also similar means or significant decreases between pairs of adjacent stages. Such results would support the assumption of a stage model and contradict that behavior change is just a continuous increase of behavior, its predictors (such as barriers, motivation, plans) and behavioral outcomes (fitness, health satisfaction).

Such analyses can be conducted by testing the trends across the means of the stage groups. Related studies testing nonlinearity patterns have provided inconsistent findings (e.g., supporting results were reported by Dohnke et al. [23]; Lippke & Plotnikoff [24]; partially supporting findings by Duan et al. [15]; Lippke et al. [19]; no support by Armitage and Arden [25]). The current study, thus, tested whether the relationship between the six FIT model stages departs from linearity for physical activity, its predictors and its outcomes.

### Testing stage assumptions: planned comparisons

The assumption of stage models posits also that an implicit ordering exists between the six stages (i.e., Not-Considering - Considering - Preparing - Fluctuating - Exploring - Maintaining), and posits that they are unequal units apart on a continuum. In other words, some adjacent stages should be significantly different whereas other adjacent stages should not be significantly different. This would indicate nonlinear patterns and support a stage model, and can be tested by a series of planned contrasts. However, with those multiple tests, an inflation of false positive findings could result, thus with planned comparisons, theoretically derived assumptions and hypotheses based on previous findings should also be tested.

An example of such an assumption is that the *PA level* is similar across non-active stages (Not-Considering, Considering, Preparing) and significantly lower than in the active stages (Fluctuating, Exploring and Maintaining). Within the active stages, a gradient increase for PA should be evident with stages continuing the goal behavior for a longer time. The assumption regarding *action plans* would be different. Here, no differences are expected within the "non-intentional" stages (Not-Considering, Considering) and within the active stages (Fluctuating, Exploring, and Maintaining). However, after setting a goal (in Preparing stage), plans are crucial. A substantial increase in contrast to the other non-active stages should therefore be evident (Not-Considering, Considering).

Another construct is *fitness*. The assumption is that the fitness level is similar in the non-active stages (Not-Considering, Considering and Exploring) and significantly lower than that in the active stages (Fluctuating, Exploring and Maintaining). Within the active stages, the fitness levels of people are expected to gradually increase the more they sporadically engage in PA (Fluctuating), in regular PA in the short term (Exploring) and in long term PA engagement (Maintaining). Based on the assumptions of the FIT model and previous relevant findings [2, 8, 12, 15, 26], the assumed differences between the stages are presented in Table 2 (Left hand side).

**Table 2:** Overview of assumptions and confirmed hypotheses for physical activity behavior

	Hypothesis (Assumption)	Findings: Confirmation of hypothesis
Physical activity behavior		
Strenuous and moderate activities (energy expenditure)	NC = C = P < F < E < Ma [2, 39]	√ √ √ √ √
Strenuous, moderate, and mild activities (energy expenditure)	NC = C = P < F < E < Ma [2, 39]	√ √ √ √ √
Social-cognitive variables		
Barriers (R)	NC > C = P > F > E > Ma [8, 40; 41]	— — √ √ √
Intrinsic motivation	NC = C < P < F = E < Ma [2, 42]	— √ √ √ √
Plans	NC = C < P = F = E = Ma [2, 24, 29]	— √ √ √ √
Health outcome		
Fitness	NC = C = P < F < E < Ma [16, 17]	— — √ — √
Health satisfaction	NC = C = P < F = E < Ma [8, 43]	√ √ √ √ —
Match of assumptions and confirmed hypotheses		27/35 = 77 %

Note. NC = Not-considering; C = Considering; P = Preparing; F = Fluctuating; E = Exploring; Ma = Maintaining

A mark (√) indicates confirmation, a hyphen (−) indicates disconfirmation. Physical activity behavior = accumulating 240 min/week. HEPA criteria = accumulating 240 min/week with at least moderate intensity (6.5 Kcal). R means reverse-scored: The higher the value, the more difficulties the individual perceive

To summarize, this study was designed to examine the quality of the FIT model stage assessment by examining the measurement quality and discontinuity patterns of theory-driven hypotheses in test variables. The following research questions were investigated.

Research questions

1. How is the measurement quality of the FIT stage algorithm when tested with behavior? Are there differences in terms of different behavior intensities (Strenuous and Moderate vs. Strenuous, Moderate, and Mild)? Measurement quality is measured by means of Misclassification, Sensitivity and Specificity.
2. Can the stage assumption be supported by nonlinear trends (higher ordered terms) across ordered stages?
3. When nonlinear trends exist, do they confirm the theoretical assumptions derived from previous findings and theoretical assumptions about the differences between adjacent stages (Table 2, Left-hand side)?

## Methods

### Participants

The study participants were university students from two universities in Hong Kong, China. 1,560 students were recruited from Physical Education (PE) classes with the assistance of PE lecturers (January 2013 - April 2013). 1,302 out of the recruited students submitted complete questionnaires during a PE course one-week later. Questionnaires containing missing data (over 50 % non-response) were omitted. After this was done, 1,012 valid questionnaires remained. The participants’ mean age was 19 years (SD = 1.26), ranging from 17–25 years, with 708 being female (70 %). 88.7 % of participants were freshmen while 11.3 % were sophomores. 655 (64.7 %) of the students majored in Liberal Arts, 283 (28 %) majored in Science, while 74 (7.3 %) did not provide study information. The body mass index (BMI) ranged from 14.17 to 58.44 (M = 20.38, SD = 3.01). After classifying, 252 (24.9 %) of the students were underweight (BMI < 18.5), 654 (64.6 %) had normal weight (BMI = 18.5 to 24.9), 61 (6.0 %) were overweight or obese (BMI ≥ 25), while 45 (4.4 %) did not provide BMI-related information.

The study procedures were approved by the Committee on the Use of Human & Animal Subjects in Teaching & Research (HASC) of Hong Kong Baptist University. Informed consent was provided by all participants before receiving the baseline questionnaires. Supporting data and further information can be obtained from the first author.

### Measures

All questionnaires had been well established and validated in previous studies involving adult populations in China [8, 13]. Prior to the main survey, young adults were asked about their understanding of the questionnaire to ensure the content validity of the questionnaire. Participants were first asked to give information regarding their gender, age, study year, height and weight. The following questionnaires were then presented.

#### FIT stages of change

To assess the current stage of PA, a stage algorithm was employed [2, 8]. The introductory text was stated as follows: “*PA includes activities of daily life (*e.g.*, climbing stairs, shopping on foot)* and *sport activities or exercises (*e.g.*, playing football, swimming, and fitness-training in a gym). Only think of those activities that you do with at least moderate intensity (some sweating and/or some breathlessness)”.* The participants were then asked, *“Did you engage in PA for an accumulated time of at least 240 min per week?”* followed by six statements for selection: (1) *Not-Considering*: “No, within the last year I was not and I am not thinking about starting in the future”; (2) *Considering*: “No, within the last year I was not, but I am thinking about starting soon”; (3) *Preparing:* “No, within the last year I was not, but I have just made the decision to and am planning to start”; (4) *Fluctuating:* “Yes, I did engage in physical activity as such, but not regularly in every week; (5) *Exploring*: “Yes, I did engage in physical activity as such, but for less than 12 months”; (6) *Maintaining*: “Yes, I did engage irregularly physical activity as such, for 12 months or more”.

#### Physical activity behavior

The PA level was measured using a self-reported scale [12, 13], which has been extensively validated by the work of Ainsworth et al. [27] and Brehm et al. [28]. In this scale, PA was divided into two types of activities, including sport and physical activities, as well as everyday life activities. Participants were first asked to select and report their sports and physical activities, such as competitive sport, fitness training, recreation activities and others. In addition, everyday life activities were selected and reported, such as climbing stairs, commuting by bike, going by foot (e.g., walking to library) and others. Following each of these two types of activities, participants were required to report the quantity and intensity of the activities they usually engaged in, respectively.

Six options for activity quantity were stated as (1) once per month or less (0 min/week); (2) two to three times per month (0 min/week); (3) up to 1 h per week (average 45 min/week); (4) 1 up to 2 h per week (average 90 min/week); (5) 2 up to 4 h per week (average 180 min/week); and (6) more than 4 h per week (average 300 min/week). Due to the very low activity frequency in the first two options, the converted time was considered as 0 min per week.

For intensity of activity, 3 options including mild (4 kcal/min), moderate (6.5 kcal/min) and strenuous (9 kcal/min) were used. The product of quantity (min/week) and intensity (kcal/min) was then computed to obtain energy expenditure (kcal/week) for each type of activity. Thus, the overall PA of participants was the summed energy consumption for the two types of activities. Two sum scores were then computed: strenuous and moderate activities on one hand (matching the HEPA recommendation), and strenuous, moderate, and mild activities on the other. These sum-score measures were treated as continuous variables for the trend and contrast analyses.

Additionally, activity responses were used to form binary outcomes to indicate whether individuals met recommended activity levels. In this study, such activity levels for young adults were treated as having accumulated at least 240 min/week. For strenuous and moderate PA level (HEPA criteria), the energy expenditure was 1,560 kcal/week (240 min/week * 6.5 kcal/min for moderate intensity). For strenuous, moderate and mild PA level, the energy expenditure was 960 kcal/week (240 min/week* 4 kcal/min for mild intensity). The binary activity outcomes were used in the analyses of classification of accuracy, sensitivity, and specificity (see Table 1).

#### Social-cognitive variables

*Barriers* were assessed with 15 items [12, 29]. The stem "Do the following reasons conform to your current living situation?" was followed by items such as "There are no physical activities appropriate for me"; "I don't have time to engage in PA"; "The distance between my dwelling place and the sporting site is too far" etc.. Respondents were asked to rank their answers on a seven-point scale, ranging from 1 (*not at all true*) to 7 (*exactly true*) (Cronbach's alpha = 0.91).

*Intrinsic motivation* was measured with 3 items [15, 30]. The stem "I intend to be physically active regularly within the next weeks and months, because…" was followed by items "…engaging in PA is a part of my life"; "…PA participation can bring me joy"; and "…I would like to obtain an experience which I don't want to miss out on". Answers were measured with a six-point scale, ranging from 1 *(not at all true)* to 6 *(exactly true)* (Cronbach's alpha = 0.87).

*Plans* were assessed with 5 items [15, 31]. The stem "I plan in detail…" was followed by items "…which PA I will perform"; "....when I will engage in PA"; "…where I will perform PA"; "…with whom I will perform PA"; and "…how often I will perform PA". Items were measured with a five-point scale, ranging from 1 *(not at all true)* to 5 *(exactly true)* (Cronbach's alpha = 0.89).

#### Health outcome variables

*Fitness* was measured using 20 items, which consisted of 4 sub-factors including strength, endurance, flexibility and coordination. Each factor included 5 items [2, 29, 32]. The stem "Are you able to…" was followed by the items for strength such as "…carry a heavy shopping bag (about 8 kg) up several floors"; the items for endurance such as "…go up several floors via the stairs without a break"; the items for flexibility such as "…reach the floor with your hands while sitting on a chair", and the items for coordination such as "…stand on one leg for at least 15 s without any extra assistance". Answers were assessed on a five-point scale, ranging from 1 (*cannot do that)* to 5 *(no problem)* (Cronbach's alpha = 0.88).

*Health satisfaction* was assessed using 7 items [15, 33]. Participants were asked to state their degree of satisfaction with their health status. The items were worded: "Regarding my general physical health status, I am…","Regarding my mental health status, I am…", and "Regarding my mental capacity, I am…". Items were measured with a seven-point scale, ranging from 1 *(very dissatisfied)* to 7 *(very satisfied)*. (Cronbach's alpha = 0.87).

### Data analysis

Misclassification, specificity and sensitivity were calculated by grouping participants according to their stage and according to their behavior (Table 1). To examine whether sensitivity and specificity rates were significantly different between the particular PA criteria (lower vs. higher criterion), a *Z*-test for two proportions was used. Moreover, the Area Under the Curve (AUC) was calculated as it incorporated both sensitivity and specificity into an overall measure of classification accuracy. A classifier with perfect sensitivity and specificity would have an AUC of 1. The AUCs were computed by running Receiver Operator Curves (ROC) analysis. Whether AUCs value were significantly different from the particular PA criteria (lower vs. higher criterion) was tested by a *Z*-test. Furthermore, the AUCs were converted into effect sizes *d,* with the calculation provided by DeCoster [34] ad suggested by Ruscio [35] to better interpret the sizes according to Cohen [36] (*d* = .20, small; *d* = .50, medium; *d* = .80, large).

Stage differences and discontinuity patterns (see above Testing Stage Assumptions) were examined using the two methods described above, namely trend analysis and planned contrasts - both using analyses of variance (ANOVA). Firstly, polynomial-based contrasts were used to test for nonlinear trends, that is, quadratic, cubic, forth-order, and fifth-order terms. The trends were tested with adjustments (weighted terms) for unequal sample sizes. Secondly, planned contrasts were used to test the differences between adjacent stages. Missing data was handled by pairwise deletion. All analyses were run with SPSS 22.0.

## Results

The sample had the following stage distribution for the FIT stage assessment: Not-Considering: 106 (10.5 %, NC); Considering: 380 (37.5 %, C); Preparing: 168 (16.6 %, P); Fluctuating: 167 (16.5 %, F); Exploring: 118 (11.7 %, E); and Maintaining: 73 (7.2 %, Ma). Thus, the highest proportion of the sample was Considering, while the lowest was Maintaining. Additionally, more participants were in the first three inactive stages (64.6 %) and fewer were in the three latter active stages (35.4 %) according to the stage classification.

### Sensitivity and specificity analysis

The resulting assessment qualities of the FIT stage algorithm are presented in Table 3. On average, 89 % of the participants in non-active stages (i.e., Not-Considering, Considering, Preparing) were correctly classified as being non-active, 50 % were correctly classified as being active by the strenuous and moderate behavior measure. Sensitivity and specificity were 71 and 76 % respectively.

**Table 3:** Assessment qualities of the staging algorithm in terms of physical activity measure

Physical activity	Correctly Classified…	Correctly Classified…	Sensitivity	Specificity	AUC (SE)	Effect size *d*
	As non-active	As active				
Strenuous and moderate behavior measure (energy expenditure)	89 % NC = 89 %	50 % F = 41 %	71 %	76%^a^	0.82 (.01)*_b_	1.29
	C = 91 %	E = 52 %				
	P = 86 %	Ma = 68 %				
Strenuous, moderate, and mild behavior measure (energy expenditure)	74 % NC = 76 %	84 % F = 76 %	64 %	89%^a^	0.86 (.01)*_b_	1.53
	C = 76 %	E = 92 %				
	P = 68 %	Ma = 90 %				

Note. NC = Not-considering; C = Considering; P = Preparing; F = Fluctuating; E = Exploring; Ma = Maintaining; Subscripts indicate significant differences between proportions and the AUC. AUC = area under the curve; SE = standard error (computed with receiver operator curves)

^a^ Z = 5.93, P < .001; _b_ Z (1012) = 4.42, *P* < .001

*P < .05

For the strenuous, moderate, and mild behavior, 74 % were correctly classified as being non-active, 84 % were correctly classified as being active. Sensitivity was 64 % and specificity 89 %. Comparing the sensitivity and specificity rates of the two criteria revealed that specificity was significantly higher when mild behavior was included (*Z* = 5.93, *p* < .001). Sensitivity was marginally higher only when strenuous and moderate behaviors were considered (Z = 1.9, *p* = .058). Behavior AUCs were between 0.82 (*SE* = .01) and 0.86 (*SE* = .01). The AUC was significantly higher when mild behavior was additionally included in comparison to when only strenuous and moderate behaviors were regarded, Z (1012) = 4.42, *P* < .001. According to the effect sizes *d*, the measurement qualities appeared to be high (between *d* = 1.29 and 1.53).

### Testing for nonlinearity

Discontinuity patterns were examined by means of polynomial-based contrast analyses using analyses of variance (ANOVA). If only linear trends are observed, this can be interpreted as supporting a pseudo-stage model; if higher-order trends are present in addition to linear trends, this indicates discontinuity [25]. Therefore, polynomial contrast analyses were used to test for nonlinear trends: quadratic, cubic, fourth-order term, and fifth-order terms.

For physical activity, 2 tests (using sum of energy expenditure for Strenuous and Moderate vs. Strenuous, Moderate, and Mild PA) were performed. For social-cognitive predictors of activity, 3 tests (Barriers, Intrinsic Motivation, and Plans) were performed. For health outcome of activity, 2 tests (Fitness and Health Satisfaction) were performed. Overall, all 7 tests resulted in a significant linear term but also required one or more higher ordered terms. This indicates that the linear term explained the most variance but residual variance could be explained by higher ordered terms (see Table 4).

**Table 4:** Results from Trend Analyses testing Linear, Quadratic, Cubic, Furth-order and Fifth-order Terms

Test Variables	Linear term F =	Quadratic term F =	Cubic term F =	Fourth-order F =	Fifth-order F =
Physical Activity					
Strenuous and moderate activities (energy expenditure)	436.20***	20.06***	3.29	17.71***	54.54***
Strenuous, moderate, and mild activities (energy expenditure)	573.65***	16.05***	7.23**	19.81***	62.96***
Social-cognitive variables					
Barriers	219.68***	1.06	0.06	16.71***	17.27***
Intrinsic motivation	240.98***	6.28*	3.68	12.37***	6.00**
Plans	141.67***	29.67***	3.80	11.57**	0.79
Health outcome					
Fitness	171.77***	0.50	0.76	7.16**	1.66
Health satisfaction	90.88***	1.87	2.67	3.18	10.04**

Note

* *P* < .05

** *P* < .01

*** *P* < .001

### Planned contrasts between stages

The means of the stage groups and the results of the planned contrast between adjacent stages are reported in Table 5.

**Table 5:** Means and Standard Deviations (in Parentheses) of the Stage Groups, Results From Planned Pair Comparisons (*n* = 956–1,012)

	Stage Groups						
Test variables	NC (*n* = 106)	C (*n* = 380)	P (*n* = 168)	F (*n* = 167)	E (*n* = 118)	Ma (*n* = 73)	Pair comparisons
	M (SD)	M (SD)	M (SD)	M (SD)	M (SD)	M (SD)	
Physical Activity (*n* = 1,012)							
Strenuous and moderate activities (energy expenditure)	487.57 (769.86)	452.47 (637.45)	608.75 (745.57)	1429.30 (1116.73)	1739.40 (946.29)	2412.50 (1412.32)	NC = C = P < F < E < Ma
Strenuous, moderate, and mild activities (energy expenditure)	568.51 (766.67)	558.89 (646.57)	776.61 (777.16)	1704.50 (1065.29)	2020.60 (960.24)	2661.60 (1288.05)	NC = C = P < F < E < Ma
Social-cognitive variables							
Barriers (*n* = 989)	3.85 (0.95)	3.75 (0.86)	3.47 (0.88)	3.07 (0.86)	2.80 (0.95)	2.33 (0.86)	NC = C > P > F > E > Ma
Intrinsic motivation (*n* = 1,001)	3.19 (1.02)	3.62 (0.84)	4.02 (0.76)	4.24 (0.93)	4.44 (0.77)	4.84 (0.94)	NC < C < P < F = E < Ma
Plans (*n* = 1,003)	2.48 (0.87)	2.89 (0.68)	3.36 (0.59)	3.31 (0.80)	3.39 (0.73)	3.53 (0.85)	NC < C < P = F = E = Ma
Health outcome							
Fitness (*n* = 956)	3.32 (0.72)	3.48 (0.58)	3.70 (0.53)	3.88 (0.55)	3.91 (0.48)	4.20 (0.47)	NC < C < P < F = E < Ma
Health satisfaction (*n* = 1,009)	3.67 (0.84)	3.64 (1.02)	3.76 (1.15)	4.22 (1.03)	4.38 (1.09)	4.62 (1.11)	NC = C = P < F = E = Ma

Note. NC = Not-considering; C = Considering; P = Preparing; F = Fluctuating; E = Exploring; Ma = Maintaining; M = Means of stage groups; SD = Standard Deviations of means for the stage groups; *n* = group size (different for the various test variables because of missing values)

#### Behavior (energy expenditure)

All the predicted significant differences across the stages for PA level were found for both measures (strenuous and moderate activity; strenuous, moderate and mild activity) (see Table 2 and 5). Individuals in Not-Considering and Considering stages, as well as those in Considering and Preparing stages reported, on average, the same levels. Moreover, persons in Fluctuating stage reported a significantly higher level of PA than those in Preparing stage. Sequentially, as stages increased from Fluctuating to Maintaining, the PA level gradually increased reaching the highest level in Maintaining stage. This is in accordance with the hypotheses (see Table 2).

For strenuous and moderate PA level (HEPA criteria), the energy expenditure was 1,560 kcal/week; and on the mean level it revealed that from a public health perspective, only individuals assigned to the Exploring or maintenance stages were sufficiently active (Table 5). For strenuous, moderate and mild PA level, the energy expenditure was 960 kcal/week; and on the mean level only individuals assigned to fluctuation, Exploring or maintenance stage were sufficiently active (Table 5).

#### Social-cognitive variables

Significant differences between adjacent stages were found in barriers (Considering vs. Preparing; Preparing vs. Fluctuating; Fluctuating vs. Exploring; Exploring vs. Maintaining), intrinsic motivation (Not-Considering vs. Considering; Considering vs. Preparing; Preparing vs. Fluctuating; Exploring vs. Maintaining), and plans (Not-Considering vs. Considering; Considering vs. Preparing) (see Table 5). The hypotheses were partially matched by these findings (see Table 2).

#### Health outcome

Significant differences between adjacent stages were found in fitness (Not-Considering vs. Considering; Considering vs. Preparing; Preparing vs. Fluctuating; Exploring vs. Maintaining) and health satisfaction (Preparing vs. Fluctuating) (see Table 5). The hypotheses were partially confirmed by these findings (see Table 2).

## Discussion

According to the literature, stage-matched treatments for physical activity promotion can only be effective if stages are adequately assessed. This study therefore attempted to provide evidence for the validity of the six FIT stage model and its assessment among university students, who are in a critical transition period. For the first time, this FIT stage algorithm was evaluated with comprehensive and rigorous examination approaches.

From a public health perspective, it was found that for strenuous and moderate PA level (HEPA criteria) with an energy expenditure of 1,560 kcal/week, only individuals assigned to Exploring stage or Maintaining stage were sufficiently active. For strenuous, moderate and mild PA level -where an energy expenditure of 960 kcal/week was regarded as sufficient- individuals assigned to the Fluctuating, Exploring or Maintenance stages were sufficiently active. This is an important finding speaking in favor of including the Fluctuating stage to appreciate individuals performing important moderate behavior. The finding can also support the accurate detection of those not adhering with the public health HEPA recommendation and are in need of interventions, as these individuals consider themselves as being sufficiently active.

Based on the AUCs, which combine sensitivity and specificity into one outcome, the findings shows the FIT stage algorithm has a suitable diagnostic accuracy with regards to PA behavior [37]. Furthermore, both AUCs were significant and indicate good measurement quality (with *d* higher 0.80 [36]). However, within the two PA level criteria, the stage algorithm is more accurate with a lower PA level than when it is compared to a higher one. Thus, our findings validate results previously found in a study with cardiac rehabilitation patients [19].

In comparison to previous studies on physical activity [19, 20, 38], sensitivity was on the average level, and specificity was comparatively high. It was additionally examined whether specificity and sensitivity were different with regard to different PA intensities. The results were partially inconsistent with those reviewed by Nigg [20] and studies by Lippke [19, 21], that is, in terms of higher sensitivity when the PA criterion was limited to strenuous and moderate level only. In other words, the FIT stage algorithm is sensitive with the HEPA criterion compared to with the lower PA criterion. This is reasonable because the behavior criterion stated in the stage assessment was set to PA accumulated with strenuous and moderate activities only. Thus, the criteria for stage assessment as well as for the behavior measure should be selected accordingly. This was, however, not true for specificity.

The results showed that specificity was significantly lower with the higher PA criterion. If the higher PA criterion is considered, it may be possible that more participants who are misclassified as being inactive by the stage algorithm are actually those who already reach the recommendation, but who perceive themselves as still aim to do so. This is an important finding because it shows the discrepancy between behavior and self-perception and that individuals need support in appreciating their achievements in order to maintain them well, especially because we know how important mastery experience is for future behavior.

Other strategies of determining the quality of the stage measures were to test nonlinearity of indicative variables across stages as well as to test hypothesized pair comparisons. With that, mean patterns were investigated. All 7 indicative variables presented higher-order trends in addition to the linear terms including quadratic, cubic, fourth-order and fifth-order terms. This clearly supports the stage assumption. However, as the linear trend was strongest in all test variables strongest, it also indicates that behavior change consists of both linear and some nonlinear processes. This is crucial to acknowledge because it supports stage assumptions while not contradicting the continuous model of behavior change.

In terms of pair contrasts, 27 out of 35 predictions were confirmed (see Table 2). In particular, for energy expenditure with the two PA intensity criteria, all 10 hypotheses were supported. For social-cognitive variables, 11 out of 15 mean differences emerged as predicted. Additionally, this study addressed the indicators of health outcomes and found 6 out of 10 predictions were supported. These analyses provided a significant contribution to current research on stage classification research. This means that apart from social-cognitive antecedents of behavior, it may be valuable to employ health outcomes as behavioral consequences to test a stage’s validation. This approach could be found in other studies as well [8, 17].

In general, with the 77 % match between hypotheses and findings (27/35), the results are promising regarding the FIT stage assessment. These comparisons were based on theory and prior research (e.g., [2, 8, 15–17, 21, 39–43]). Thus, the findings underline the relevance of the previous stage models -because they served to develop evidence-based predictions- and the FIT model which was developed on basis of adding a new stage to the previous models. The results indicate that more than 3/4 predictions were found in this sample of university students, and that the FIT model could be advantageous because of its new stage. Appreciating that students in Fluctuation stage perform the recommended behavior is a more optimistic view than lumping Fluctuation with inactive stages together and treating them as students with no behavioral experiences and disregarding potential resources such as previous mastery experience [7].

According to the FIT stage model, the Fluctuating stage falls between the Preparing and Exploring stages [2]. Individuals in this stage perform HEPA behavior often but irregularly: They may be doing sufficient PA for three consecutive weeks, then lowering the quantity of PA or stopping PA altogether for one or two weeks, and subsequently resuming HEPA behavior again for the next two weeks. Thus, compared to Preparing stage where individuals do not adhere to the HEPA criteria, people in Fluctuating stage expend more energy, perceive less barriers (e.g., with more confidence to do PA, finding time to start PA), possess stronger intrinsic motivation (e.g., competition, enjoyment) to engage in PA, and gain more in health outcomes from performing PA. In comparison to Exploring stage, where individuals regularly engage in HEPA for less than 1 year, people in Fluctuating stage are at a higher risk for disengagement and they expend less energy, perceive more barriers (e.g., lacking coping strategy to prevent distraction), and obtain less in health outcomes from regularly doing PA. Most of these assumptions regarding Fluctuating stage could be supported by the present findings (see Table 2). Some results are also consistent with a previous study involving a sample with Chinese and German adults [8].

Thus, this work is noteworthy and important because it shows that behavior change is not a simple on-off process but rather a dynamic process. It also indicates that behavior change may depend on other environmental influences such as study strain, and work overload at some times, with more relaxed at other times.

One *limitation* of the current study is that the majority of sample in this study consisted of female students (70 %). In addition, more students were in inactive stages (NC, C or P) compared to active stages (F, E or Ma), which might influence the results of stage assessment. Therefore, it would be worth seeking other samples with more balanced gender and activity distributions in the future. In addition, 290 questionnaires with a non-response rate of over 50 % were excluded from data analysis, which might influence the representation of study results. Moreover, reliance on the self-reporting of PA is another limitation. Although the validation of the PA behavior measure employed in this study was well established [27, 28] and used in other studies before [2, 8, 11, 17], objective measures of PA would be a stronger test and may help to improve the validation of stage assessment.

## Conclusions

In conclusion, the current study shows the potential usefulness of the FIT stage model and its respective algorithm in university student samples. In the future, this version can be adapted for other age populations (e.g., teenagers, adults, old adults) according to the variety of HEPA recommendations. Moreover, in order to draw more powerful inferences on stage validation and public health implications, further studies should examine the stages, stage movements and health behavior change by using stage-tailored interventions.
